# Explaining variation in GP referral rates for x-rays for back pain

**DOI:** 10.1186/1748-5908-1-15

**Published:** 2006-08-02

**Authors:** Rachel Baker, Jan Lecouturier, Senga Bond

**Affiliations:** 1 School of Population and Health Sciences, University of Newcastle upon Tyne, Newcastle upon Tyne, UK

## Abstract

**Background:**

Despite the availability of clinical guidelines for the management of low back pain (LBP), there continues to be wide variation in general practitioners' (GPs') referral rates for lumbar spine x-ray (LSX). This study aims to explain variation in GPs' referral rates for LSX from their accounts of the management of patients with low back pain.

**Methods:**

Qualitative, semi-structured interviews with 29 GPs with high and low referral rates for LSX in North East England. Thematic analysis used constant comparative techniques.

**Results:**

Common and divergent themes were identified among high- and low-users of LSX. Themes that were similar in both groups included an awareness of current guidelines for the use of LSX for patients with LBP and the pressure from patients and institutional factors to order a LSX. Differentiating themes for the high-user group included: a belief that LSX provides reassurance to patients that can outweigh risks, pessimism about the management options for LBP, and a belief that denying LSX would adversely affect doctor-patient relationships. Two specific differentiating themes are considered in more depth: GPs' awareness of their use of lumbar spine radiology relative to others, and the perceived risks associated with LSX radiation.

**Conclusion:**

Several key factors differentiate the accounts of GPs who have high and low rates of referral for LSX, even though they are aware of clinical guideline recommendations. Intervention studies that aim to increase adherence to guideline recommendations on the use of LSX by changing the ordering behaviour of practitioners in primary care should focus on these factors.

## Background

Low back pain (LBP) is a global and increasing problem [1]. Estimates of point prevalence range between 12% and 35% and lifetime prevalence between 49% and 80% [2]. The cost of LBP in the United Kingdom is high, both to the NHS (National Health Service) and in terms of the wider societal costs [2,3]. While there are a number of serious conditions that cause LBP, most LBP is non-specific, benign, and self limiting, although it may become recurrent [4,5]. Non-specific LBP is classified by the duration of symptoms into acute (less than 3–6 weeks), sub-acute (less than three months) and chronic (more than three months) [6-8]. One distinction between the acute and chronic forms of non-specific LBP is that the prognosis for the former is reported to be generally good [9]. For most people with acute LBP, symptoms rapidly improve within one month and continue to do so for up to three months, but from then on any residual symptoms of pain and disability remain constant [6,10]. The majority will experience at least one recurrent episode in the subsequent 12 months, and around 5% of those with acute lower back pain will develop chronic LBP [7].

The relationship between x-ray findings and non-specific LBP is unclear [11,12], and despite a link between radiological findings of degenerative disorders and LBP [11-13] such findings have little implication for the management of LBP. Recent trials have shown that radiography for primary health care patients with LBP has no effect on health outcomes, although patient satisfaction is higher [14,15]. Lumbar spine x-ray is associated with a dose of ionising radiation equivalent to approximately 65 chest x-rays [16]. Therefore, unnecessary examinations should be avoided.

The use of lumbar spine x-ray (LSX) in cases of LBP is not routinely indicated. Degenerative changes commonly detected by LSX are non-specific, and the main value of LSX, according to guidelines from the Royal College of Radiologists, is for young people (<20) where spondylolisthesis or ankylosing spondylitis are a concern, or for those older than 55. In the presence of specific 'red flag' symptoms, such as sphincter or gait disturbance, or widespread neurological deficit, Magnetic Resonance Imaging (MRI) is the preferred investigation [16]. Despite clinical guidelines [16] there is wide variation in the use of plain film x-rays for patients with LBP from primary care [17,18], and many requests do not conform to guideline recommendations [19,20].

Studies aiming to find reasons for GPs requesting LSX for patients with LBP have relied on quantitative methods (e.g., [21-23]) and have shown that GP requests for LSX derive from both health and non-health factors. Maintaining patient satisfaction with their care and offering reassurance to patients are often important. These studies, whilst informative, have not explored in-depth the rationales for referral behaviour. Two qualitative studies have been identified, both using focus group methods. The first, undertaken in the United States [24]., sought to explain the negative findings of an intervention study to improve referral practice. The second, a Norwegian study, identified factors affecting decisions to order spine radiography focussing on the barriers to guideline adherence [25]. No UK studies that seek to understand GP referral behaviour and explain different LSX referral practices for LBP have been found. This study aims to investigate reasons for GP referral for LSX for patients presenting with LBP and explain observed differences in referral rates. It is based on the premise that GPs with high and low referral rates for LSX will give different accounts of their perceptions, experience and management of LBP and LSX.

## Methods

The study was conducted among GPs in the North East of England during 2000. The number of LSXs requested by individual GPs in the preceding year was obtained from radiology departments in three hospitals. Absolute frequencies of LSX requests were adjusted to take account of working hours of GPs and list sizes, and the sample was rank-ordered according to the adjusted referral frequency. GPs were sampled sequentially from the high and low ends of the distribution. They were contacted by letter and subsequently by telephone inviting them to take part in a single interview lasting between 60 and 90 minutes and were reimbursed for their time at locum rates. Recruitment continued until categories were saturated. In addition, five GPs in research practices were interviewed first and provided comments on the interview and topic guide. No significant revisions were made as a result, and they are included in the analysis. Of 55 GPs who were invited to take part, 29 (53%) agreed and their characteristics are described in Table 1. Twenty six chose to be interviewed at their practice premises, two chose to be interviewed at home, and one at the university.

**Table 1:** Characteristics of 29 GPs interviewed

Gender	Male	24
	Female	5
Frequency of Use of LSX	High	14
	Low	15
Type of Area	Urban	26
	Rural	3
Years since qualified	<20 years	17
	>20 years	12
Working hours	Full Time	24
	Part Time	5
		
Practice Type (Training)	Training practice	16
	Not training practice	13
List Size	< 5,000	6
	5 to 10,000	10
	10,000 over	13
Number of Partners	Single Handed	2
	2 to 5 partners	12
	> 5 partners	15
GP Trainer	GP trainer	9
	Not trainer	20
Area	Research Practice *	5
	Newcastle upon Tyne	7
	Teesside	9
	Gateshead	8

*Geographical area for research practices are not shown for reasons of anonymity.

Interviews were conducted by RB (researcher), who remained blind during the interview to whether the GPs were categorised as high- or low-users of LSX (from radiology records). Interviews were informed by a topic guide, and were tape recorded and transcribed verbatim. Field notes [26] were dictated to tape after interviews and were transcribed. Emerging concepts and themes were recorded in a research diary [27]. The topic guide comprised four main sections: Section 1 was concerned with GP practice and locality characteristics; Section 2 aimed to elicit information about GPs' perceptions of patients presenting with LBP; and Section 3 focussed on how GPs handled specific cases of LBP, and they were asked in advance to retain the case notes of recent patients to discuss their histories, consultations, and decisions made. This case-based approach allowed probing of actual decisions to refer for LSX or choosing other courses of action. In Section 4, GPs were asked specifically about their beliefs and attitudes towards the use of LSXs. The order of topics in the interviews varied depending on spontaneous discussion and the emphasis accorded to topics by respondents. The topic guide was amended throughout the study as new themes were identified using constant comparative analysis.

A sub-sample of five transcripts was fully coded by two researchers (RB, SB) to develop an initial coding frame and to identify concepts and themes. Subsequent themes were debated and agreed upon by all three authors. Drawing on principles of constant comparison [28], the development of the coding frame and emergent themes were subject to deviant case analysis. NVivo qualitative analysis software [29] was used to index and interrogate the data. Themes that were consensual across high- and low-users of LSX were classified as 'convergent themes' and views which differentiated the two groups were classified as 'divergent themes.'

Ethical approval to conduct the study was obtained from local research ethics committees in Newcastle and North Tyneside, Gateshead and North Tees. All interviewees were assured anonymity in any reports or publications of the findings.

## Results

### Convergent themes

A number of convergent themes that related to the decision to request an LSX and showed no pattern of association with high- or low-users of LSX were identified. These were broadly categorised into three groups: 'clinical,' 'psycho-social,' and 'institutional' factors. GPs were knowledgeable about the existence and general thrust of clinical guidelines for the management of LBP, could articulate their main messages, and did not challenge their content. Some discussed general issues around the problems with adherence to guidelines in a clinical context, but these GPs did not take issue with the specific recommendations of either the Royal College of General Practitioners back pain guidelines [30] or the Royal College of Radiologist guidelines [16].

*Yeah, I'm happy that the Royal College of Radiologists are telling us that there's little point in x-raying backs. Very rarely do we need to x-ray backs really if we're worried about a disc it's no good – needs an MRI, certainly nothing to x-ray a back with less than six weeks history, and it's only rarely perhaps that that is the best investigation. For some of the more concerning problems, you might pick up bony mets' but you're going to get a raised ESR... GP14, high*.

*I think the protocol is there to protect us. I think we've got good guidelines from both radiology and from the Royal College of GPs' Guidelines on management of low back pain. GP16, low*.

The content of the available guidelines went unchallenged and the limitations of x-rays as a diagnostic tool for LBP were acknowledged. GPs were sensitive to the difficulties they and their patients encountered in dealing with chronic back pain.

Important social factors that influenced referral decisions included patient expectations and the pressure on GPs to 'do something.' Experience of patient pressure was often stated in strong terms and illustrated with examples:

*We explained that to him but he eventually came with a big brother and sort of insisted he was going to be x-rayed. So he was x-rayed and told he had minor degenerative changes, and now I know from the consultation this morning he has made a formal complaint .. Litigation. GP10 high*.

An x-ray which was deemed to be 'negative' (in terms of excluding certain diagnoses) was seen by some respondents as providing reassurance to patients, although this was qualified by some respondents who acknowledged that clinically insignificant findings on x-ray may raise anxiety due to continued uncertainty, rather than reduce it. Patient anxiety over LBP as a symptom of something more serious was an important influence, and LSXs were used to allay fears, particularly in relation to cancer. GPs were also influenced by the social and economic issues of importance to people with LBP:

*We know the families so if there are marital problems we're aware of it, if they are having problems at work we're usually aware of it, if they don't like work and want to get out of it we're aware of it, if they've been involved in a road traffic accident recently we're aware of it. So there's lots of issues and it does help to colour – you know – it does fill in the background, and it does help you decide whether or not this patient needs to be referred urgently or whether we sit and wait and watch GP13 low*.

All of the GPs faced difficulties caused by waiting lists for referral to secondary care, and access to magnetic resonance imaging (MRI) was limited compared with easy and quick access to LSX. The ideal investigation or treatment pathway was not always available.

*They've got an 8 month wait for a back problem so... surely somewhere along the way they're going to be saying shouldn't we have some x-rays done. You've got to say 'yes we could get those done so that they are ready when you see the specialist.' And it actually buys more time, it gives another reason for the patient to, you know, feel well, there's something else happening. Which is atrocious really." GP16 low*.

In summary, both high- and low-users of LSX were aware of the guidelines with respect to LBP and LSX, spoke about the pressures created by waiting times for secondary care or MRI, experienced patient pressure for something to be done and their anxieties (especially about LBP as heralding cancer), and were aware of social and socio-economic factors and the diagnostic limitations of LSX.

### Divergent themes

Divergent themes were those more prevalent or given much higher degree of emphasis in either the high- or low-referral group.

Obtaining a 'negative' LSX result as a legitimate means to reassure patients that their LBP did not stem from serious pathology was a view more strongly associated with the accounts of high-user GPs.

*We are well aware of the College of Radiologists telling us we are being terribly irresponsible x-raying all these people with normal lumber spines, but it has a ... it has a ... a very reassuring effect with patients. GP21 High*.

Low-users expressed more qualms about the outcome of such attempts at reassurance and described a more complex view of the effects of LSX findings that may be misleading, misinterpreted by patients or doctors, or insufficient to overcome patients' fears.

*I mean if you do tests you inevitably get small abnormalities which aren't reassuring, so if you're going to reassure someone you reassure them 100% or you don't ... or you're not reassuring. So you know, if you do an x-ray and say, 'Oh there's some small changes but that's wear and tear' and everyone ... people say that, erm how reassuring is that? GP22 Low*.

Views of the management options available to them and their success were similarly divided. GPs in the high-user group emphasised a pessimistic account of the options available to them and the negative prognosis for chronic back pain sufferers.

*The ones that are sort of chronic sort of pain that no one can do anything about because it's a problem with analgesia, nothing really works very well. GP27 High*.

A more positive outlook for LBP was found in GPs in the low referral group, who talked about a greater repertoire of approaches to patient management, even when the condition was chronic.

*I don't think any, in any of the forms of back pain that, that I ever feel that... you know, there's nothing at all that can be done for the person or that there's nothing at all that they can do. GP 03 Low*.

The need to preserve the doctor-patient relationship was an important factor for some GPs and was at times influential in their decisions to investigate and manage LBP. In the high-user group there was greater emphasis on the fragility of this relationship and concern over the detrimental consequences of it breaking down. LSX was used, at times, to meet patient demand in decisions that were inappropriate in strict clinical terms, in order to preserve relationships with patients.

*But at the end of the day you give in, cause it's not worth it, it's not worth losing a patient doctor relationship unless you want to. GP 24 High*.

Low-users of LSX were similarly aware of the threat to the doctor-patient relationship by refusing to x-ray. They knew that patients who were not satisfied might 'shop around' to find a GP willing to comply. However, they were prepared to face these eventualities as part of the give and take of what they regarded as 'being a good GP.'

### Concern about radiation

Concern about exposure to radiation was emphasised, often in strong terms, by most of the GPs in the low-referral group.

*I am very anti X-ray. I see that X-ray has a dose of radiation associated [with it] and with back X-rays gives out a significant amount of radiation, and really I think a lot of people, particularly in casualty departments and GPs, don't take that into account and it's really important. GP06, low*.

GPs who referred more patients for LSX were far less concerned about exposure to radiation. They explained their lack of concern in three ways. Firstly, the absolute dose of radiation was perceived to be minimal compared with other procedures such as computed tomography (CT) or barium studies. Secondly, the perceived benefits of the x-ray, including patient and GP reassurance, greatly outweighed the perceived radiation risks, and thirdly, relative to other treatment decision risks such as drug prescribing, the risk of exposing a patient to LSX radiation was regarded as small.

Such reasoning was couched in terms of their own experience and the absence of *visible *ill effects of x-rays on patients or NHS staff.

Interviewer: Do you have any concerns about using x-ray?

*Respondent: No, no. I mean there's, the so-called radiation hazard but you know I've been in this game long enough now, and it depends on how many x-rays you actually do. We don't do all that many. GP15, high*.

*I can't say I'm convinced that there is... good evidence that patients are actually coming to harm by having x-rays... Having worked with a lot of people in the operating theatres where x-rays are being sprayed around, and I'm not aware of any of them coming to major harm. Okay, we used to wear lead most of the time – but not all the time. GP24, high*.

Several GPs in the low group also were unconcerned about radiation risk. They were influenced in their decisions not to refer by other potent factors including: financial cost, the dubious clinical benefit of x-ray for LBP, and an awareness of the value of adhering to guideline recommendations.

*I don't really worry about x-rays. I mean, I think you know we are talking about people who might have one or two or three x-rays in their lifetime. I don't usually worry about that in terms of their health. I mean my main concern is... the resource issue really. GP09, low*.

### Knowledge of own use of x-ray

GPs from the low group were aware of and judged their relative use of LSX more accurately than those in the high group. Low-users had developed other ways of managing patients with LBP, and this was likely to be a feature of the practice as well as of individual GPs.

*I rarely do lumbar x-rays but we don't, probably haven't done two in the last year, and one of those was as the request of the physio. GP16, low*.

Nine of the 14 GPs who were relatively high-users of LSX stated that they were either unsure or were low-users of LSX. Despite their relatively frequent use of LSXs, they often perceived their use as infrequent.

*I don't x-ray people's backs much at all really because they're not much use are they really – x-rays. GP27, high*.

## Discussion

Variation in the use of LSX in cases of LBP in primary care remains a problem. Before interventions can be designed to change behaviour and reduce variation there needs to be an in-depth understanding of current behaviour [31]. This study identified a number of themes which are common to, and which distinguish the accounts of, GPs with high and low referral rates for LSX, enabling a better understanding of GPs' decision making in this context. In this analysis we have created a picture of an archetypal high LSX user and have identified different exemplary characteristics of a low LSX user through GPs' own descriptions of their practices and beliefs.

There are, however, some limitations to this study that should be acknowledged. Since these interviews were carried out there have been changes in the organisation of UK primary health care, e.g., Practice-Based Commissioning (PBC), which may or may not have an impact on these findings. Whilst it is unlikely that initiatives such as PBC will have an effect on GPs' perceptions of LBP, clinical guidelines, LSX and their patients, such as are reported in this study, institutional and structural changes in the NHS may affect GPs' views of the treatment options available to them. This study cannot offer any insight into the possible effects of these recent changes. In addition, a purposive sampling strategy was used and respondents were selected on the basis of their levels of LSX use. As such, the sample is not representative and nothing can be said about the distribution of these themes in the population.

In terms of the application of our findings, two principal divergent themes have been identified that could be targeted in specific and simple interventions: firstly, GPs in the high-use group perceived their own use of LSX to be relatively low, and secondly, they had low levels of concern about the risks of radiation associated with LSX. That GPs are unaware of their relatively high use of x-ray suggests that informing them of their use relative to others may reduce the number of LSXs amongst this high group. However, intervention studies investigating the effect of feedback on referral rates for LSX have shown this strategy to be ineffective in changing behaviour [18,32].

The differing concerns between high- and low-users of LSX surrounding radiation risk may be of greater relevance to the design of future intervention studies. The importance of this theme also is substantiated by a recent qualitative study of the prescription of new drugs by GPs [33]. Using a similar comparative design the authors report 'attitudes to risk perception and benefit' amongst the key dimensions that classify high- and low-prescribers of new drugs and state that, "High-prescribers were more inclined to underplay the risks of new drugs, so freeing them to prescribe a new drug they believed offered therapeutic effectiveness.." p590. This study suggests that GPs who request relatively frequent x-rays for LBP differ from their colleagues in their assessment of radiation risk. It is possible, therefore, that these GPs may change their behaviour in response to information about the radiation risks associated with LSX, and revisit their assessment of the costs and benefits of requesting such examinations. This hypothesis provides an avenue for future research.

## Abbreviations

LSX: Lumbar spine X-ray.

LBP: Low Back Pain.

MRI: Magnetic Resonance Imaging.

CT: computed tomography.

## Competing interests

The author(s) declare that they have no competing interests.

## Authors' contributions

RB conducted all of the interviews, devised the coding frame, analysed the data, and drafted the paper. RB, SB and JL contributed to the development of the topic guide, coding frame, and emergent themes throughout data collection and concurrent analysis. SB designed the study and coded a sub-sample of interview transcripts. SB and JL redrafted the paper and commented on subsequent drafts. SB is guarantor for this project. All authors read and approved the final manuscript.
